# Author Correction: Symbioses shape feeding niches and diversification across insects

**DOI:** 10.1038/s41559-023-02137-2

**Published:** 2023-07-03

**Authors:** Charlie K. Cornwallis, Anouk van ’t Padje, Jacintha Ellers, Malin Klein, Raphaella Jackson, E. Toby Kiers, Stuart A. West, Lee M. Henry

**Affiliations:** 1 Department of Biology, Lund University, Lund, Sweden; 2 Amsterdam Institute for Life and Environment, section Ecology and Evolution, Vrije Universiteit, Amsterdam, the Netherlands; 3 Laboratory of Genetics, Wageningen University and Research, Wageningen, the Netherlands; 4 School of Biological and Behavioural Sciences, Queen Mary University of London, London, UK; 5 Department of Biology, University of Oxford, Oxford, UK

**Keywords:** Social evolution, Coevolution, Evolutionary ecology

Correction to: *Nature Ecology& Evolution* 10.1038/s41559-023-02058-0. Published online 18 May 2023.

In the version of this article originally published, there was an error in the Fig. 1c *x*-axis label order: “Phloem” and “Blood,” in the second and third positions, respectively, were accidentally reversed. The labels have been corrected in the HTML and PDF versions of the article.

