# Supplementary material for: Symbioses shape feeding niches and diversification across insects
Source: Nat Ecol Evol. 2023 May 18;7(7):1022–44. doi: 10.1038/s41559-023-02058-0 (PMC10333129; doi:10.1038/s41559-023-02058-0)
Supplement: Supplementary file 1 [file 41559_2023_2058_MOESM1_ESM.pdf]

nature portfolio

Corresponding author(s): Cornwallis, C. K. Henry, L. M.

Last updated by author(s): Feb 26, 2023

# Reporting Summary

Nature Portfolio wishes to improve the reproducibility of the work that we publish. This form provides structure for consistency and transparency in reporting. For further information on Nature Portfolio policies, see our Editorial Policies and the Editorial Policy Checklist.

## Statistics

For all statistical analyses, confirm that the following items are present in the figure legend, table legend, main text, or Methods section.

| n/a | Confirmed | |
|---|---|---|
| ☐ | ☒ | The exact sample size (*n*) for each experimental group/condition, given as a discrete number and unit of measurement |
| ☐ | ☒ | A statement on whether measurements were taken from distinct samples or whether the same sample was measured repeatedly |
| ☐ | ☒ | The statistical test(s) used AND whether they are one- or two-sided<br>*Only common tests should be described solely by name; describe more complex techniques in the Methods section.* |
| ☐ | ☒ | A description of all covariates tested |
| ☐ | ☒ | A description of any assumptions or corrections, such as tests of normality and adjustment for multiple comparisons |
| ☐ | ☒ | A full description of the statistical parameters including central tendency (e.g. means) or other basic estimates (e.g. regression coefficient) AND variation (e.g. standard deviation) or associated estimates of uncertainty (e.g. confidence intervals) |
| ☐ | ☒ | For null hypothesis testing, the test statistic (e.g. *F*, *t*, *r*) with confidence intervals, effect sizes, degrees of freedom and *P* value noted<br>*Give P values as exact values whenever suitable.* |
| ☐ | ☒ | For Bayesian analysis, information on the choice of priors and Markov chain Monte Carlo settings |
| ☐ | ☒ | For hierarchical and complex designs, identification of the appropriate level for tests and full reporting of outcomes |
| ☐ | ☒ | Estimates of effect sizes (e.g. Cohen's *d*, Pearson's *r*), indicating how they were calculated |

*Our web collection on statistics for biologists contains articles on many of the points above.*

## Software and code

Policy information about availability of computer code

| | |
|---|---|
| Data collection | All data was complied using the open source software R. All versions of the R packages used together with the code and data are available at the open science framework: DOI 10.17605/OSF.IO/TYK7C. |
| Data analysis | All data analysis was performed using the open source software R and BayesTraits V4. All versions of the R packages used together with the code and analysis outputs are available at the open science framework: DOI 10.17605/OSF.IO/TYK7C. |

For manuscripts utilizing custom algorithms or software that are central to the research but not yet described in published literature, software must be made available to editors and reviewers. We strongly encourage code deposition in a community repository (e.g. GitHub). See the Nature Portfolio guidelines for submitting code & software for further information.

## Data

Policy information about availability of data

All manuscripts must include a data availability statement. This statement should provide the following information, where applicable:

- Accession codes, unique identifiers, or web links for publicly available datasets
- A description of any restrictions on data availability
- For clinical datasets or third party data, please ensure that the statement adheres to our policy

R code, BayesTraits code, data and analysis results are available at the open science framework: DOI 10.17605/OSF.IO/TYK7C. Full citations of references in supplementary tables are given in the method references48-382.

## Human research participants

Policy information about studies involving human research participants and Sex and Gender in Research.

| | |
|---|---|
| Reporting on sex and gender | NA |
| Population characteristics | NA |
| Recruitment | NA |
| Ethics oversight | NA |

Note that full information on the approval of the study protocol must also be provided in the manuscript.

# Field-specific reporting

Please select the one below that is the best fit for your research. If you are not sure, read the appropriate sections before making your selection.

☐ Life sciences ☐ Behavioural & social sciences ☒ Ecological, evolutionary & environmental sciences

For a reference copy of the document with all sections, see nature.com/documents/nr-reporting-summary-flat.pdf

# Ecological, evolutionary & environmental sciences study design

All studies must disclose on these points even when the disclosure is negative.

| | |
|---|---|
| Study description | We show that the evolution of obligate symbiosis with microbes has played a key role in explaining the adaptive radiation of insects. Using phylogenetic comparative analyses of data on 1850 microbe-insect symbioses across 402 insect families we show that:<br>• Obligate symbiosis has allowed insects feeding on generalist diets to unlock specialized resources such as phloem, xylem, blood and wood.<br>• Across diverse niches, insect feeding specialization is explained by symbiotic microbes supplying a single key nutrient - B vitamins.<br>• By allowing the exploitation of new feeding niches, symbionts have shaped insect diversification. In some cases, such as herbivorous insects, symbionts have facilitated spectacular radiations. In other cases, such as blood feeding, feeding specialization has been severely limited diversification. |
| Research sample | The study analyses published accounts of 1850 microbe-insect symbioses. Full details of the species studied and the methods used are given in the supplementary data tables 1-4 and in the method references 101-409. |
| Sampling strategy | Published literature was searched to retrieve all studies examining microbe-insect symbiosis. Full details of how literature was found are given in the Material and Methods. |
| Data collection | We complied a database on insect-microbe symbioses by: (1) searching published literature using the following key words [order name] OR [family name] AND “symbio”* using the search engines Web of Science and Google scholar during 2015-2017 and again in 2020, (2) searching several prominent reviews (e.g. Ries 1931, Schneider 1939, Müller 196243, Buchner 1965, Douglas 1989, Abe et. al. 2000, Bourtzis and Miller 2003, 2006 and 2009, Baumann 2005, Baumann et. al. 2013), and (3) forward and backward searches from the resulting papers. A full list of the papers screened can be found in Supplementary Table 2. |
| Timing and spatial scale | The time periods of data collection are given in the Material and Methods. Published literature was searched during 2015-2017 and updated in 2020 due to the time taken for data analyses to be completed. |
| Data exclusions | The aim of our paper was to investigate the evolution of beneficial obligate symbioses. We therefore excluded studies: (1) on parasitic symbionts, such as those that manipulate host reproduction (e.g. Spiroplasma, Cardinium, Wolbachia) that have not evolved beneficial functions; (2) that failed to screen the entire insect (e.g. only performed insect gut analyses); and (3) on symbionts with presumed beneficial functions, but that lacked data needed for our obligate criteria (see below). Fungal and protist symbionts were included where data on host dependency was available. Analyses of host-symbiont coevolution were restricted to symbionts for which a phylogeny could be constructed (bacteria with 16S rRNA genetic data: see section 2 for details). |
| Reproducibility | All data analysis was performed using the open source software R and BayesTraits V4. All versions of the R packages used together with fully reproducible R project scripts are available at the open science framework: DOI 10.17605/OSF.IO/TYK7C. |
| Randomization | NA. The study was a comparative analysis of all published data. |
| Blinding | NA. The study was a comparative analysis of all published data. |

Did the study involve field work? ☐ Yes ☒ No

# Reporting for specific materials, systems and methods

We require information from authors about some types of materials, experimental systems and methods used in many studies. Here, indicate whether each material, system or method listed is relevant to your study. If you are not sure if a list item applies to your research, read the appropriate section before selecting a response.

## Materials & experimental systems

| n/a | Involved in the study | |
|---|---|---|
| ☒ | ☐ | Antibodies |
| ☒ | ☐ | Eukaryotic cell lines |
| ☒ | ☐ | Palaeontology and archaeology |
| ☐ | ☒ | Animals and other organisms |
| ☒ | ☐ | Clinical data |
| ☒ | ☐ | Dual use research of concern |

## Methods

| n/a | Involved in the study | |
|---|---|---|
| ☒ | ☐ | ChIP-seq |
| ☒ | ☐ | Flow cytometry |
| ☒ | ☐ | MRI-based neuroimaging |

## Animals and other research organisms

Policy information about studies involving animals; ARRIVE guidelines recommended for reporting animal research, and Sex and Gender in Research

| | |
|---|---|
| Laboratory animals | The study did not involve laboratory organisms |
| Wild animals | The study analyses published accounts of 1850 microbe-insect symbioses. Full details of the species studied and the methods used are given in the supplementary data tables 1-4 and in the method references 101-409. |
| Reporting on sex | NA |
| Field-collected samples | NA |
| Ethics oversight | NA |

Note that full information on the approval of the study protocol must also be provided in the manuscript.
